# Longitudinal associations of occupational cumulative noise exposure duration and hearing loss with lipid profiles: a 9-year cohort study

**DOI:** 10.3389/fpubh.2026.1964407

**Published:** 2026-09-25

**Authors:** Pan Liu, Huixia Ji, Ye Chen, Dandan Liu

**Affiliations:** 1 Outpatient Department, Nanjing Prevention and Treatment Center for Occupational Diseases, Nanjing, Jiangsu, China; 2 Department of Occupational Disease, Nanjing Prevention and Treatment Center for Occupational Diseases, Nanjing, Jiangsu, China

**Keywords:** binaural high-frequency average hearing threshold, longitudinal study, occupational noise exposure, total cholesterol, triglycerides

## Abstract

**Background:**

Occupational noise exposure causes auditory damage and is also closely associated with systemic metabolic abnormalities. The impact of long-term noise exposure on longitudinal lipid changes remains unclear.

**Objective:**

This longitudinal study examined the association of baseline cumulative noise exposure duration with serum triglycerides (TG) and total cholesterol (TC) in male workers, and further examined whether binaural high-frequency average hearing threshold (BHFTA), an objective marker of noise-induced cochlear damage, modified longitudinal lipid trends.

**Methods:**

In this retrospective longitudinal cohort study, 431 male workers were followed for 9 years. Occupational noise exposure was defined according to the Chinese national standard GBZ/T 224-2010 as exposure to an 8-hour equivalent continuous A-weighted sound level of at least 80 dB(A) (Lex, 8h), and was operationalized as the cumulative duration of employment in noise-exposed positions ascertained from plant administrative records. Linear mixed-effects models were used to analyze the effects of follow-up time, baseline cumulative noise exposure duration (mean-centered, time-invariant), and their interaction on natural log-transformed TG and TC, adjusted for age, body mass index, lipid-lowering medication use, smoking, and alcohol consumption. Simple slopes were estimated at representative exposure durations (3, 10, and 20 years). Because individual biological noise dose could not be directly measured, BHFTA was used as an objective marker of accumulated biological dose and individual susceptibility in sensitivity analyses, with simple slopes estimated at BHFTA values of mean ± 1 standard deviation (SD).

**Results:**

Baseline cumulative noise exposure duration was significantly and positively associated with serum TG levels, with each additional year of exposure corresponding to an approximately 2.0% higher TG level (β = 0.0203 per year on ln(TG), 95% CI: 0.0029–0.0377, *P* = 0.022), whereas its association with TC did not reach statistical significance (*P* = 0.077). The follow-up time and baseline exposure duration interaction was negative in direction, that is, the annual rate of lipid increase tended to be smaller among workers with longer baseline exposure; this reached borderline significance for TG (β = −0.0013, 95% CI: −0.0027–0.0001, *P* = 0.066) and statistical non-significance for TC (*P* = 0.479). The estimated annual TG increase displayed a monotonic descriptive gradient across baseline exposure duration of 3, 10, and 20 years (5.35%, 4.39%, and 3.03% per year, respectively). In sensitivity analyses, BHFTA interacted positively with follow-up time for both outcomes (TG: 0.0023, 95% CI: 0.0013–0.0034; TC: 0.0005, 95% CI: 0.0001–0.0009); the estimated annual increase rose from 2.07 to 4.61% for TG and from 0.73 to 1.28% for TC as BHFTA increased from mean – 1 SD to mean + 1 SD (14.3–24.9 dB).

**Conclusions:**

Baseline cumulative noise exposure duration was associated with elevated TG levels, and the attenuated rate of lipid increase over time may partly reflect a healthy worker survivor effect. In contrast, BHFTA may be more sensitive than administrative exposure records in revealing the metabolic consequences of chronic noise exposure. Hearing conservation and metabolic monitoring should be integrated into occupational health surveillance.

## Introduction

Globally, occupational noise exposure is a major health concern (1). Occupational noise exposure not only causes irreversible hearing loss but is also closely linked to multi-system health damage (2). Several studies have shown an independent association between noise exposure and metabolic syndrome (3). In cross-sectional studies, noise exposure is linked to elevated triglycerides (TG), low high-density lipoprotein cholesterol (HDL-C), and abnormal fasting glucose levels and glycated hemoglobin (4). In addition, noise exposure is associated with higher levels of cholesterol biomarkers such as low-density lipoprotein cholesterol (LDL-C) and intermediate-density lipoprotein cholesterol (IDL-C) and with changes in unsaturated fatty acid levels (5). Animal experiments reveal that chronic noise activates the CRTC2/CREB-PCK1 and SREBP1/SCD signaling pathways, which regulate hepatic gluconeogenesis and lipid synthesis, respectively, via the gut-liver axis (6). These lipid disturbances are of important clinical significance. Dyslipidemia is a major risk factor for cardiovascular disease, and noise exposure has been shown to be associated with increased cardiovascular mortality (7). Atherosclerosis, endothelial dysfunction, oxidative stress, and cardiovascular events such as coronary heart disease and stroke are associated with noise exposure (8, 9). Therefore, clarifying the longitudinal association between noise exposure and lipid profiles is important for understanding and preventing noise-related cardiovascular disease.

However, most existing evidence comes from cross-sectional studies focusing on the direct association between cumulative noise exposure and metabolic indicators (3, 4). While longitudinal studies have linked nighttime traffic noise to cholesterol biomarkers (5), such as LDL-C and IDL-C, and unsaturated fatty acids, occupational noise research is limited. Long-term longitudinal data on occupational noise and lipid trends are lacking. In particular, few studies have used hearing loss as a indicator of accumulated auditory impairment from cumulative noise exposure.

Using a 9-year longitudinal cohort, this study examined the association of occupational noise exposure duration with longitudinal changes in TG and total cholesterol (TC). In addition, the binaural high-frequency average hearing threshold (BHFTA) was used as an indicator of noise-related hearing impairment, and the associations of cumulative noise exposure levels with lipid changes were compared with those of this noise-related early biological effect indicator. This study aims to provide more comprehensive longitudinal evidence for the metabolic health risk assessment of occupational noise exposure and a scientific basis for developing targeted occupational health interventions.

## Methods

### Study design and population

Data were derived from routine occupational health examinations of workers at a vehicle manufacturing plant. In this retrospective longitudinal cohort study, the baseline examination was conducted in 2016, with follow-up examinations in 2018, 2021, 2023, and 2025; all participants had a history of occupational noise exposure. The participant selection process is summarized in Figure 1. Briefly, among the 804 workers examined at baseline, female workers (*n* = 16) were excluded owing to the small sample size; we further required a baseline cumulative noise exposure of at least 3 years and valid baseline serum TG and TC measurements (*n* = 67 excluded). Of the remaining eligible workers, 431 had valid TG and TC measurements at both baseline (2016) and the final follow-up (2025) and had remained in noise-exposed positions throughout the study period, while 290 workers (40.2% of the eligible pool) were excluded because they had left noise-exposed positions during follow-up or lacked valid endpoint lipid data. Requiring valid measurements at both endpoints ensured that intra-individual trajectories could be observed and that the nine-year progression trends could be reliably estimated. Because eligibility required remaining employed in noise-exposed positions throughout the study period and having valid TG and TC measurements at both baseline and the final follow-up, the study population consists of “survivors” who remained continuously exposed to noise over the nine-year period; workers who retired, resigned, died, or were transferred away from noise-exposed positions during follow-up are not represented. The analyzed cohort may therefore be healthier than the totality of workers ever exposed (survivor effect). The study was conducted in accordance with the Declaration of Helsinki (as revised in 2024). The study protocol was approved by the Ethics Committee of Nanjing Prevention and Treatment Center for Occupational Diseases (Approval No. 2025-005), and the requirement for written informed consent was waived owing to the retrospective nature of the study. Before data analysis, all personal identifiers were removed, and each subject was assigned a unique anonymous identification code to ensure privacy and confidentiality.

**Figure 1:**
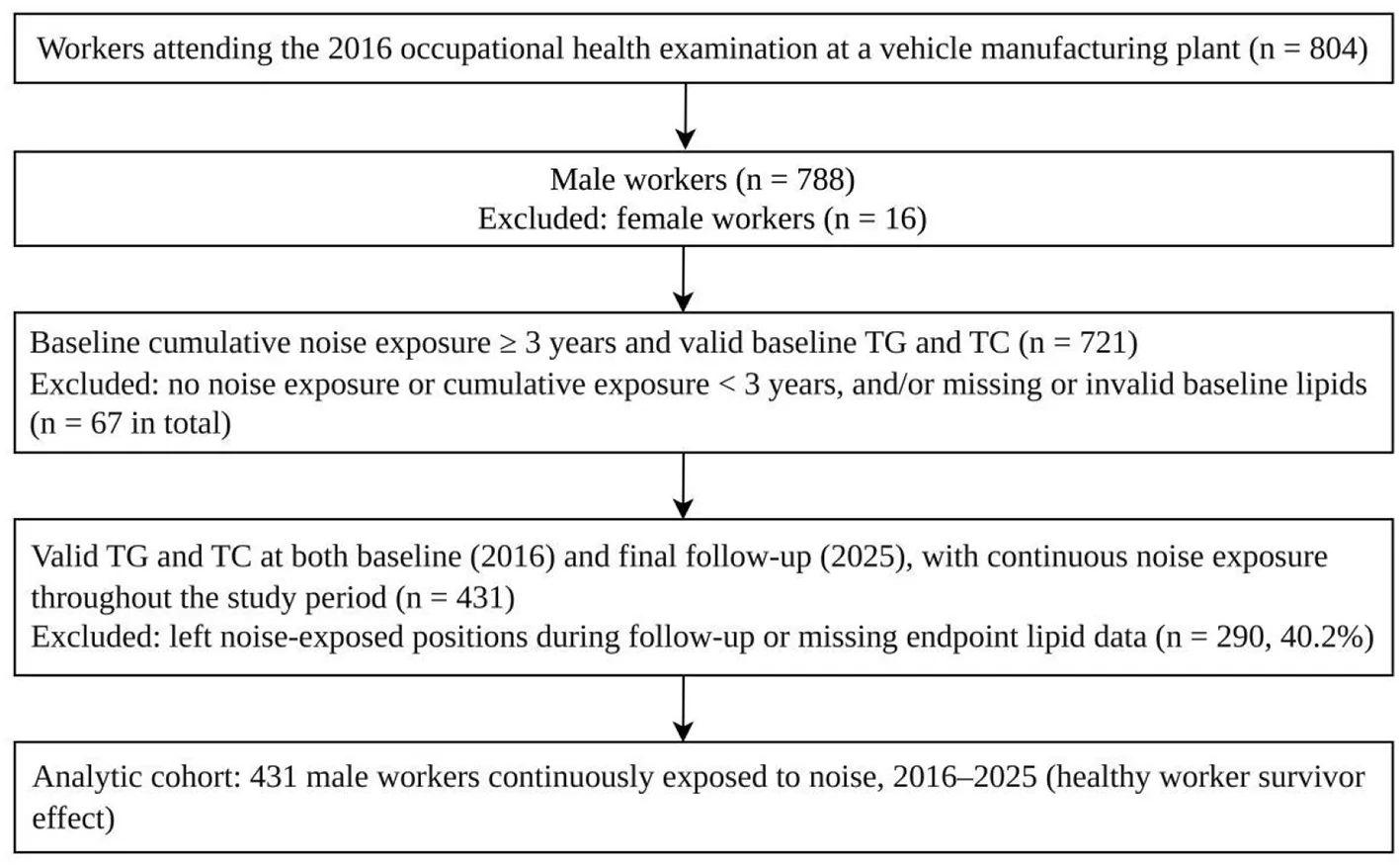
Participant selection and attrition in the longitudinal study cohort, 2016–2025. Among 804 workers attending the 2016 baseline occupational health examination, 16 female workers were excluded owing to the small sample size; a further 67 workers were excluded because they had no noise exposure or a baseline cumulative exposure < 3 years and/or missing or invalid baseline lipid data. Of the remaining 721 eligible workers, 431 had valid serum triglyceride (TG) and total cholesterol (TC) measurements at both baseline (2016) and the final follow-up (2025) and had remained in noise-exposed positions throughout the study period; the remaining 290 (40.2%) had left noise-exposed positions during follow-up or lacked valid endpoint lipid data. The analytic cohort therefore consists of “survivors” continuously exposed to noise over the nine-year period. Valid lipid measurements were available for 431, 399, 408, 367, and 431 participants in 2016, 2018, 2021, 2023, and 2025, respectively (2,036 observations in total, all of which entered the mixed-effects models). Missingness was intermittent: participants with missing data at one wave partly returned at subsequent waves. TG, triglycerides; TC, total cholesterol.

### Data collection

Occupational noise exposure was defined as exposure to a noise level of 80 dB(A) or higher (Lex, 8h). Chinese national standard GBZ/T 224-2010 defines noise operations as work involving sound detrimental to hearing, health, or other hazards, where the equivalent sound level of noise exposure is ≥80 dB(A) for 8 h/d or 40 h/week. The plant's administrative department, which maintains the personnel and occupational health records of all employees, ascertained employment in noise-exposed positions as defined above and provided, for each worker, the cumulative duration (in years) of such employment; the value reported for the baseline examination was taken as the baseline cumulative noise exposure duration. No job-title histories or workshop-level noise measurement records were included in the data provided. An inquiry was used to obtain information about smoking status, alcohol consumption, and the use of lipid-lowering medications (statins or fibrates). On the day of the physical examination, weight and height were measured. After an overnight fast, blood samples were collected for lipid analysis. Serum TG levels were measured using the glycerol phosphate oxidase method (10). TC levels were determined using the cholesterol oxidase method (11). During the same examination session, BHFTA were measured.

### Definitions of variables

The primary outcomes were longitudinal trends in serum TG and TC, each entered into models as natural log-transformed values to approximate normality and stabilize variance; in Table 1, TG, TC, and baseline cumulative exposure duration are summarized as medians (IQR) owing to their skewed distributions, and other continuous variables as mean ± standard deviation (SD).

**Table 1:** Baseline (2016) characteristics of the study population and longitudinal trends of lipid profiles.

Baseline characteristics	ALL (*N* = 431)	
Age (years)	32.7 ± 5.5	
BMI (kg/m^2^)	24.8 ± 3.1	
Smoking status		
No	203 (47.1%)	
Yes	228 (52.9%)	
Alcohol drinking		
No	366 (84.9%)	
Yes	65 (15.1%)	
Lipid-lowering treatment		
No	427 (99.1%)	
Yes	4 (0.9%)	
Cumulative exposure duration (years)	15.0 (13.0, 18.0)	
BHFTA (dB)	22.4 ± 3.9	
TG (mmol/L)	1.2 (0.8, 1.7)	
TC (mmol/L)	4.4 (3.9, 4.9)	
Longitudinal lipid measurements		
Year	TG median (IQR)	TC median (IQR)
2016 (*N* = 431)	1.16 (0.80, 1.69)	4.41 (3.86, 4.89)
2018 (N = 399)	1.14 (0.75, 1.77)	4.23 (3.77, 4.82)
2021 (N = 408)	1.56 (1.00, 2.40)	4.74 (4.21, 5.29)
2023 (N = 367)	1.60 (1.10, 2.54)	5.01 (4.51, 5.66)
2025 (N = 431)	1.42 (0.93, 2.23)	4.61 (4.13, 5.18)
*P*-trend	< 0.001	< 0.001

Continuous variables are presented as mean ± standard deviation (SD) or median (interquartile range, IQR), and categorical variables as frequency (percentage). P-trend was derived from the time coefficient of unadjusted linear mixed-effects models. BMI, body mass index; BHFTA, binaural high-frequency average hearing threshold; TG, triglycerides; TC, total cholesterol.

Baseline cumulative noise exposure duration was defined as the total number of years a worker had been employed in noise-exposed positions (≥80 dB(A) Lex, 8h, per GBZ/T 224-2010) from the start of such work up to the baseline (2016) examination. It entered all main models as a continuous, time-invariant variable, mean-centered at the sample mean. BHFTA was calculated per GBZ 49-2014 as the mean threshold at 3, 4, and 6 kHz averaged across both ears (dB HL) and entered the sensitivity models as a continuous, time-varying variable, mean-centered at the mean of all audiometric measurements.

Covariates were coded as follows: age (years, continuous, time-invariant, at baseline); BMI (kg/m^2^, continuous, time-invariant, calculated as weight in kilograms divided by height in meters squared, at baseline); smoking status (binary, time-invariant: yes if ≥3 cigarettes/day for ≥6 months); alcohol consumption (binary, time-invariant: yes if drinking at least once per week during the past year); and lipid-lowering treatment (binary, time-varying: use of statins or fibrates recorded at each examination). Age was entered at baseline because a time-varying age term would be deterministically collinear with follow-up time. Complete repeated measurements of BMI, smoking status, and alcohol consumption could not be retrieved in this retrospective cohort, so baseline values were used; lipid-lowering treatment was updated at each examination because medication changes directly affect concurrent lipid levels. Follow-up time was coded as years since the 2016 baseline examination, with baseline coded as 0.

### Statistical analysis

All statistical analyses were performed using R software (version 4.3.3). Continuous variables are presented as mean ± SD or median (IQR), and categorical variables as frequency (percentage). The longitudinal trend of lipids across the five follow-up waves was estimated using an unadjusted linear mixed-effects model (LMM) with follow-up time as a fixed effect and participant-specific random intercepts, and the P for trend was derived from the coefficient of time to test for a linear trend over the follow-up period.

### Main analysis: cumulative noise exposure duration and lipid trends

Linear mixed-effects models (LMMs) were constructed using the lmerTest package to examine the association of baseline cumulative noise exposure duration with lipid progression. The outcome variables were natural log-transformed TG and TC. The primary predictors were follow-up time (years since baseline, 2016, with baseline coded as 0), baseline cumulative noise exposure duration (a time-invariant characteristic), and their interaction term. Age, BMI, smoking status, alcohol consumption, and lipid-lowering therapy (a time-varying covariate) were adjusted for in all models, and random intercepts for each participant were incorporated to account for the correlation of repeated measurements within individuals. Baseline exposure duration was mean-centered at the sample mean, whereas follow-up time was retained on its original scale; this parameterization avoids the structural multicollinearity that would arise from defining cumulative exposure as baseline duration plus follow-up time, and allows the main effect of time to be interpreted as the annual lipid change at the mean baseline exposure duration. Variance inflation factors (VIFs) were computed for all predictor variables (follow-up time, baseline cumulative noise exposure duration, their interaction, and covariates); all VIFs were below 1.2, indicating no problematic multicollinearity. Marginal R^2^ and conditional R^2^ were calculated using the performance package to quantify the variance explained by the fixed effects and by the full model, respectively. To characterize the pattern of the follow-up time × exposure duration interaction, simple slopes of log-transformed TG were estimated at representative baseline exposure durations (3, 10, and 20 years) using the emmeans package, and the annual rate of change in lipid levels was calculated as [exp(slope) – 1] × 100%; because the corresponding interaction for TC was not statistically significant, simple slopes were not estimated for TC.

### Sensitivity analysis: BHFTA and lipid changes

Because administratively recorded exposure duration reflects external exposure opportunity and may not capture the biologically effective noise dose, and given the healthy worker survivor effect, the LMMs were refitted with BHFTA as the exposure indicator. BHFTA was regarded as an objective physiological marker of accumulated auditory impairment that integrates the biologically effective noise dose reaching the inner ear with individual susceptibility, rather than a direct measure of occupational noise exposure. Among the 431 workers, 1,726 observations with concurrent audiometric measurements were available. LMMs with the same covariate structure were fitted, with follow-up time retained on its original scale and BHFTA mean-centered, and an interaction term between BHFTA and follow-up time was included. Simple slopes were estimated at BHFTA values corresponding to the mean ± 1 SD (14.3, 19.6, and 24.9 dB), and annual percentage changes were calculated as described above. A two-sided *P*-value less than 0.05 was considered statistically significant.

## Results

### Baseline characteristics of the study population and longitudinal trends of lipid profiles

This longitudinal analysis included 431 male workers. Table 1 shows the baseline characteristics and longitudinal lipid measurements of the study population. Participants were aged 32.7 ± 5.5 years at the baseline. The mean BMI was 24.8 ± 3.1 kg/m^2^. Among the participants, 52.9% were current smokers and 15.1% consumed alcohol. Only 0.9% took lipid-lowering medications. The median baseline cumulative noise exposure was 15.0 years (13.0, 18.0). From 2016 to 2025, both TG and TC levels showed a significant increasing trend over time (*P*-trend < 0.001 for both).

### Primary analysis: association of baseline cumulative noise exposure duration with lipid levels and trends

Linear mixed-effects models were constructed to assess the association of baseline cumulative noise exposure duration with lipid levels and their progression over time. Natural log-transformed TG and TC served as dependent variables. Follow-up time (years since baseline), baseline cumulative noise exposure duration, and their interaction term were included. The models adjusted for age, BMI, smoking history, and alcohol history, and the time-varying covariate of lipid-lowering treatment was also included. To mitigate structural multicollinearity, baseline noise exposure duration was mean-centered, whereas follow-up time was retained on its original scale (0 = baseline visit). As shown in Table 2, baseline exposure duration was significantly and positively associated with ln(TG) at the baseline visit (0.0203 per year; 95% CI: 0.0029–0.0377; *P* = 0.022), corresponding to an approximately 2.0% higher TG level per additional year of exposure, whereas its association with ln(TC) did not reach statistical significance (0.0052 per year; 95% CI: −0.0006–0.0110; *P* = 0.077). Lipid levels also increased significantly over follow-up (0.0365 per year for ln(TG) and 0.0120 per year for ln(TC), both *P* < 0.001). The interaction between follow-up time and baseline exposure duration was negative in direction for both outcomes. For ln(TG), the interaction coefficient was −0.0013 (95% CI: −0.0027–0.0001; *P* = 0.066), indicating a borderline tendency for the annual TG increase to attenuate with longer baseline exposure. For ln(TC), the interaction coefficient was −0.0002 (95% CI: −0.0008–0.0004; *P* = 0.479).

**Table 2:** Effects of baseline cumulative noise exposure duration on lipid trends.

Outcome	Variable	Beta (95% CI)	*P*-value
ln(TG)	Fixed effects	–	–
	Intercept	−1.0946 (−1.6265, −0.5627)	< 0.001
	Follow-up time (years)	0.0365 (0.0319, 0.0411)	< 0.001
	Baseline exposure duration (years)	0.0203 (0.0029, 0.0377)	0.022
	Lipid-lowering treatment (yes)	−0.2961 (−0.4075, −0.1847)	< 0.001
	Smoking (yes)	0.1191 (0.0182, 0.2201)	0.021
	Alcohol drinking (yes)	0.1356 (−0.0049, 0.2761)	0.059
	Age (years)	−0.0084 (−0.0179, 0.0011)	0.085
	BMI (kg/m^2^)	0.0591 (0.0431, 0.0750)	< 0.001
	Follow-up × Exposure duration	−0.0013 (−0.0027, 0.0001)	0.066
	Random effects (variance)		
	Subject (ID)	0.2471	—
	Residual	0.1182	—
	Model fit		
	Marginal R^2^	0.1340	—
	Conditional R^2^	0.7197	—
ln(TC)	Fixed effects		
	Intercept	1.2008 (1.0301, 1.3716)	< 0.001
	Follow-up (years)	0.0120 (0.0102, 0.0139)	< 0.001
	Baseline exposure duration (years)	0.0052 (−0.0006, 0.0110)	0.077
	Lipid-lowering treatment (yes)	−0.1374 (−0.1815, −0.0932)	< 0.001
	Smoking (yes)	0.0150 (−0.0174, 0.0473)	0.366
	Alcohol drinking (yes)	0.0116 (−0.0335, 0.0567)	0.615
	Age (years)	0.0002 (−0.0029, 0.0032)	0.910
	BMI (kg/m^2^)	0.0102 (0.0051, 0.0153)	< 0.001
	Follow-up time × exposure duration	−0.0002 (−0.0008, 0.0004)	0.479
	Random effects (variance)		
	Subject (ID)	0.0240	—
	Residual	0.0188	—
	Model fit		
	Marginal R2	0.0656	—
	Conditional R2	0.5896	—

Outcomes were natural log-transformed. Models adjusted for age, BMI, smoking status, alcohol drinking, and lipid-lowering treatment. Marginal R^2^ represents the variance explained by fixed effects; Conditional R^2^ represents the variance explained by the combined fixed and random effects. TG, triglycerides; TC, total cholesterol; BMI, body mass index; CI, confidence interval.

Consistent with this borderline interaction, the annual growth rate of TG exhibited a monotonic downward gradient with increasing baseline cumulative noise exposure duration (Table 3 and Figure 2A). In simple-slopes analyses, the estimated annual growth rates of TG for workers with baseline noise exposure duration of 3, 10, and 20 years were 5.35% (95% CI: 3.55%−7.18%), 4.39% (95% CI: 3.53%−5.25%), and 3.03% (95% CI: 2.15%−3.91%), respectively, all of which were significantly greater than zero (all *P* < 0.001). Given the borderline statistical significance of the interaction (*P* = 0.066), this gradient should be interpreted as a descriptive trend that aligns with the main-effect association between exposure duration and TG, rather than as established effect modification. Simple slopes for TC were not estimated because the corresponding follow-up time × exposure duration interaction was not statistically significant (*P* = 0.479).

**Table 3:** Simple slope analysis of TG trends at specific baseline cumulative noise exposure duration.

Outcome	Baseline exposure duration	Estimated slope (95% CI)^*^	Annual change, % (95% CI)^†^
ln(TG)	3 years	0.0521 (0.0345, 0.0694)	5.35% (3.55%, 7.18%)
	10 years	0.0429 (0.0347, 0.0512)	4.39% (3.53%, 5.25%)
	20 years	0.0298 (0.0213, 0.0383)	3.03% (2.15%, 3.91%)

^*^Estimated slope represents the regression coefficient (β) for time on the natural log-transformed lipid levels, derived from the linear mixed-effects models including the interaction between time and cumulative exposure duration.

^†^Annual change represents the percentage change in lipid levels per year, calculated as [exp (slope)-1] × 100%.TG, triglycerides; CI, confidence interval.

**Figure 2:**
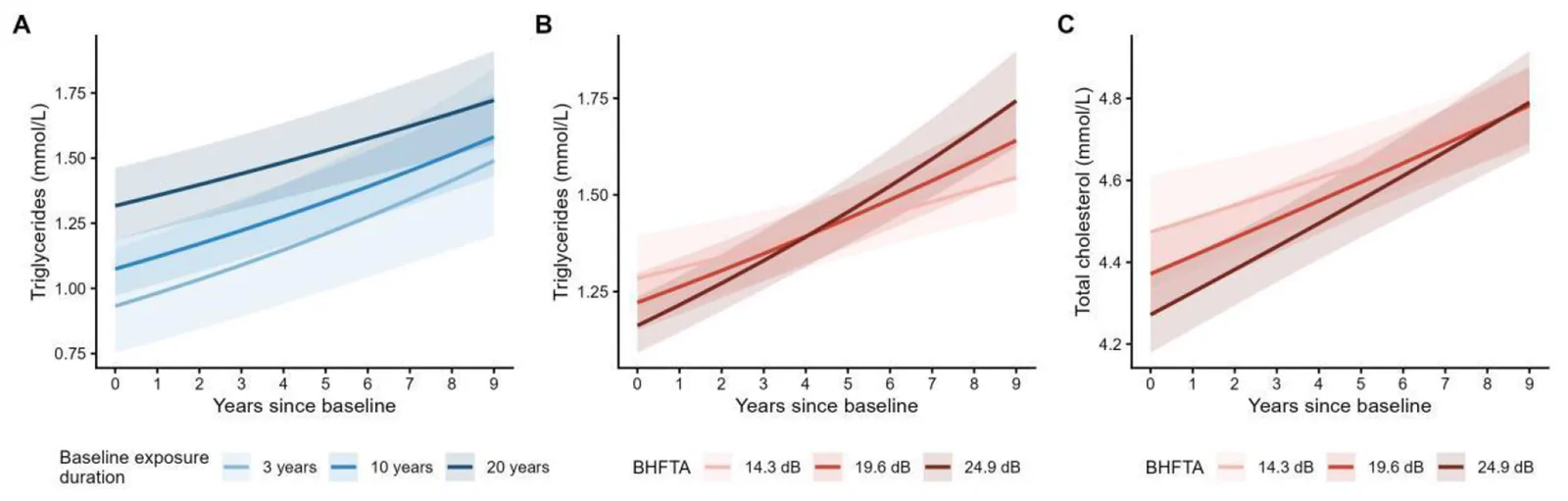
Predicted trends of serum triglycerides and total cholesterol by baseline cumulative noise exposure duration and bilateral high-frequency hearing threshold among male noise-exposed workers, 2016–2025. Predicted geometric-mean concentrations of **(A)** triglycerides (TG) by baseline cumulative noise exposure duration (3, 10, and 20 years) and of **(B)** TG and **(C)** total cholesterol (TC) by bilateral high-frequency hearing average threshold (BHFTA; 14.3 dB = mean – 1 SD, 19.6 dB = mean, and 24.9 dB = mean + 1 SD) over 9 years of follow-up. Curves are model-based estimates from linear mixed-effects models with random intercepts, adjusted for age, BMI, smoking history, alcohol history, and time-varying lipid-lowering treatment; shaded bands represent 95% confidence intervals. *P*-values shown in each panel refer to the follow-up time × moderator interaction term (**A**: *P* = 0.066; **B**: *P* < 0.001; **C**: *P* = 0.019).

### Sensitivity analysis: interaction between BHFTA and follow-up time on lipid trends

For a sensitivity analysis (*n* = 431), the LMMs were refitted with BHFTA as the exposure indicator. After adjusting for the same covariates, with follow-up time retained on its original scale and BHFTA mean-centered (mean = 19.6 dB), the interaction term between follow-up time and BHFTA had a significant positive effect on both ln(TG) (β = 0.0023, 95% CI: 0.0013–0.0034) and ln(TC) (β = 0.0005, 95% CI: 0.0001–0.0009) (Table 4).

**Table 4:** Effects of BHFTA on lipid trends in sensitivity analysis.

Outcome	Variable	Beta (95% CI)	*P*-value
ln(TG)	Fixed effects		
	Intercept	−1.1290 (−1.6768, −0.5811)	< 0.001
	Follow-up time (years)	0.0328 (0.0270, 0.0386)	< 0.001
	BHFTA (dB)	−0.0095 (−0.0173, −0.0017)	0.018
	Lipid-lowering treatment (yes)	−0.2652 (−0.3833, −0.1471)	< 0.001
	Smoking (yes)	0.1205 (0.0146, 0.2264)	0.026
	Alcohol drinking (yes)	0.1382 (−0.0094, 0.2858)	0.067
	Age (years)	−0.0068 (−0.0163, 0.0026)	0.157
	BMI (kg/m^2^)	0.0596 (0.0429, 0.0763)	< 0.001
	Follow-up time × BHFTA	0.0023 (0.0013, 0.0034)	< 0.001
	Random effects (variance)		
	Subject (ID)	0.2640	—
	Residual	0.1168	—
	Model fit		
	Marginal R^2^	0.1306	—
	Conditional R^2^	0.7333	—
ln(TC)	Fixed effects		
	Intercept	1.1738 (0.9917, 1.3558)	< 0.001
	Follow-up time (years)	0.0100 (0.0077, 0.0123)	< 0.001
	BHFTA (dB)	−0.0043 (−0.0074, −0.0012)	0.006
	Lipid-lowering treatment (yes)	−0.1365 (−0.1836, −0.0895)	< 0.001
	Smoking (yes)	0.0136 (−0.0215, 0.0488)	0.448
	Alcohol drinking (yes)	0.0042 (−0.0448, 0.0532)	0.867
	Age (years)	0.0010 (−0.0022, 0.0041)	0.542
	BMI (kg/m^2^)	0.0107 (0.0051, 0.0162)	< 0.001
	Follow-up time × BHFTA	0.0005 (0.0001, 0.0009)	0.019
	Random effects (variance)		
	Subject (ID)	0.0273	—
	Residual	0.0191	—
	Model Fit		
	Marginal R^2^	0.0591	—
	Conditional R^2^	0.6131	—

Outcomes were natural log-transformed. Models adjusted for age, BMI, smoking status, alcohol drinking, lipid-lowering treatment. BHFTA was used as a continuous biomarker of actual hearing loss severity.

Marginal R^2^ represents the variance explained by fixed effects; Conditional R^2^ represents the variance explained by the combined fixed and random effects. TG, triglycerides; TC, total cholesterol; BHFTA, binaural high-frequency average hearing threshold; BMI, body mass index; CI, confidence interval.

Simple slope analysis (Table 5 and Figures 2B, C) showed a monotonic graded positive association between BHFTA and the annual rate of lipid increase. For TG, the estimated annual growth rate rose from 2.07% (95% CI: 1.18%−2.97%) at 14.3 dB (mean – 1 SD), to 3.33% (95% CI: 2.73%−3.94%) at 19.6 dB (the mean), and to 4.61% (95% CI: 3.84%−5.39%) at 24.9 dB (mean + 1 SD). For TC, the corresponding annual growth rate increased from 0.73% (95% CI: 0.38%−1.09%) to 1.01% (95% CI: 0.77%−1.24%) and to 1.28% (95% CI: 0.98%−1.58%) at the same three BHFTA levels.

**Table 5:** Simple slope analysis of lipid trends at specific BHFTA.

Outcome	BHFTA (dB)	Estimated slope (95% CI)^*^	Annual change, % (95% CI)^†^
ln(TG)	BHFTA = 14.3 dB	0.0205 (0.0117, 0.0293)	2.07% (1.18%, 2.97%)
	BHFTA = 19.6 dB	0.0328 (0.0270, 0.0386)	3.33% (2.73%, 3.94%)
	BHFTA = 24.9 dB	0.0451 (0.0377, 0.0525)	4.61% (3.84%, 5.39%)
ln(TC)	BHFTA = 14.3 dB	0.0073 (0.0037, 0.0108)	0.73% (0.38%, 1.09%)
	BHFTA = 19.6 dB	0.0100 (0.0077, 0.0123)	1.01% (0.77%, 1.24%)
	BHFTA = 24.9 dB	0.0127 (0.0098, 0.0157)	1.28% (0.98%, 1.58%)

^*^Estimated slope represents the regression coefficient (β) for time on the natural log-transformed lipid levels, derived from the linear mixed-effects models including the interaction between time and binaural high-frequency average hearing threshold (BHFTA). The selected thresholds (14.3, 19.6, and 24.9 dB) represent representative levels of hearing loss severity.

^†^Annual change represents the percentage change in lipid levels per year, calculated as [exp(slope)-1] × 100%.TG, triglycerides; TC, total cholesterol; BHFTA, binaural high-frequency average hearing threshold; CI, confidence interval.

## Discussion

This study examined the association of occupational noise exposure with longitudinal trends in lipid metabolism through a nine-year follow-up of 431 male noise-exposed workers, with five examinations conducted between 2016 and 2025. In the main analysis, baseline cumulative noise exposure duration was significantly and positively associated with TG levels; its interaction with follow-up time, although negative in direction, reached only borderline significance for TG, suggesting a descriptive tendency for lipid increases to attenuate with longer exposure, a pattern plausibly related to the survivor effect. In the sensitivity analysis, when the models were refitted with BHFTA as the exposure indicator, an objective marker of accumulated auditory impairment, the interaction with follow-up time turned significantly positive for both TG and TC, with greater hearing damage associated with a faster rate of lipid increase.

Previous studies on the metabolic effects of occupational noise often relied on job titles or workplace noise records, were mostly cross-sectional, and lacked longitudinal follow-up (3, 12). In large-scale occupational epidemiology, precise individual noise doses are difficult to obtain because of constraints on cost, feasibility, and worker compliance. In the present study, BHFTA was additionally analyzed as a marker of accumulated auditory impairment. BHFTA is a sensitive functional indicator of noise-induced cochlear damage, and previous studies have shown that it correlates with cumulative noise exposure (13–15). However, BHFTA is not a direct measure of occupational noise exposure. Elevated thresholds partly reflect functional impairment of the outer hair cells and are jointly determined by actual exposure intensity and duration, use of hearing protection, individual susceptibility, and repair capacity, and may also be influenced by non-occupational noise and ototoxic factors.

In the main analysis, baseline cumulative noise exposure duration was significantly and positively associated with TG levels, with each additional year of exposure corresponding to an approximately 2.0% higher TG level, whereas its association with TC did not reach statistical significance. Consistent with our findings, Zhang et al. reported a non-linear association between occupational noise exposure duration and dyslipidemia, with higher risk among workers exposed to 75–85 dB(A) for 11–24.5 years (16). Mechanistically, noise may act as a chronic stressor that activates the hypothalamic–pituitary–adrenal (HPA) axis and sympathetic nervous system, sustaining catecholamine and cortisol release and promoting inflammation and oxidative stress (12). Meanwhile, the interaction between follow-up time and baseline exposure duration was negative in direction for both outcomes, reaching borderline significance for TG, suggesting that workers with longer exposure duration tended to exhibit a slower rate of lipid increase over time. The possible explanation for this phenomenon is the survivor effect. The survivor effect is a common source of selection bias in long-term follow-up studies in epidemiology (17). This study included workers with at least 3 years of occupational noise exposure, and these workers continued to be exposed for up to 9 years. In such a long-term design, workers who remained in the workforce may have had greater metabolic compensation capacity and individual tolerance. In contrast, workers who were more susceptible to noise and had weaker metabolic function may have left their jobs or been transferred early because of health problems, and thus did not contribute to later follow-up (18, 19). Such selective retention may have led to underestimation of the observed association between exposure and outcome, particularly among long-term exposed workers. In addition, older workers with longer service may have paid more attention to diet and daily habits due to occupational health interventions, which may partly offset the lipid increase associated with aging and prolonged noise exposure. This study found only borderline significance for the interaction between follow-up time and baseline exposure duration. This trend may be partly attributable to the limited sample size, or may reflect the survivor effect or other unmeasured factors.

A sensitivity analysis using BHFTA revealed a positive interaction effect. In this study, BHFTA, reflecting accumulated auditory impairment, was associated with systemic lipid metabolism disorders. The two conditions may share noise-induced pathophysiology. Noise activates the HPA axis and the sympathetic nervous system. This activation induces systemic oxidative stress and a chronic low-grade inflammatory state. The systemic inflammatory environment disrupts vascular endothelial function and promotes lipid metabolism disorders (20, 21). Also, it affects inner ear microcirculation, causing sensorineural hearing loss. Additionally, remnant cholesterol (RC) is a cholesterol component within triglyceride-rich lipoproteins. Beyond conventional components, it reflects atherogenic and inflammatory burdens. Previous studies have shown that RC levels are significantly correlated with the severity of hearing loss (22). Hearing threshold damage may be linked to lipid metabolism disorders by noise-induced systemic inflammation. Moreover, noise exposure can regulate gut microbiota and serum metabolites. It triggers the SREBP1/SCD signaling pathway of lipid synthesis, thereby causing lipid metabolism disorders (6).

Notably, the two exposure indicators yielded discordant interaction directions. The follow-up time interaction of administratively recorded exposure duration was negative in direction and only borderline significant, whereas the follow-up time interaction of BHFTA was significantly positive for both outcomes. Several explanations should be considered. First, administrative duration indicates external exposure opportunity rather than the dose actually absorbed. This measurement error, together with the possible survivor effect described above, may bias the time interaction of duration toward or below the null. Second, BHFTA is downstream of exposure. It reflects accumulated cochlear damage from all noise sources, including non-occupational exposure, as well as individual susceptibility, and is therefore not specific to occupational noise. Third, workers with the same nominal exposure duration may differ considerably in absorbed dose and susceptibility, and BHFTA captures this heterogeneity. The discordance between the two indicators may thus indicate that administrative records fail to capture the biologically effective dose, that BHFTA reflects factors beyond occupational noise, or both. These possibilities cannot be disentangled in the absence of individual noise dosimetry, which underscores the value of combining external exposure records with biological effect markers in future studies.

In the main analysis, although longer baseline cumulative noise exposure duration was associated with higher TG levels, the follow-up time and baseline exposure duration interaction was negative in direction and reached only borderline significance, indicating that lipid deterioration over time was not significantly accelerated by administratively recorded exposure duration. In contrast, in the sensitivity analysis, BHFTA interacted significantly and positively with time for both TG and TC, indicating that workers with higher hearing thresholds experienced a faster rate of lipid increase. These findings suggest that hearing thresholds, as a marker of accumulated biological damage, may be more informative than administratively recorded exposure duration in identifying workers whose lipid metabolism worsens over time. This is consistent with Jin et al., who reported a dose–response association between BHFTA and hypertension risk in a large cross-sectional survey, supporting the interpretation of BHFTA as a marker of cumulative noise-related auditory burden (23).

Comparison of model fit between the two lipids showed that the marginal R^2^ of the TG model (0.1340) was higher than that of the TC model (0.0656), suggesting that the environmental, demographic, and lifestyle factors included in the models explained a larger share of the variation in TG than in TC. The likely reason for this difference is that TG is highly sensitive to sympathetic activation and to lifestyle factors such as smoking, whereas TC levels are less responsive to lifestyle changes and environmental exposure and are primarily influenced by genetic background (24, 25). For both lipids, the conditional R^2^ values were substantially higher than the corresponding marginal R^2^ values (0.7197 for TG and 0.5896 for TC), indicating that most of the longitudinal variation was attributable to stable between-individual differences captured by the random effects rather than to the measured covariates.

This study has several limitations. Individual noise intensity and dose were not monitored during follow-up, so a precise individual cumulative noise dose could not be obtained. In addition, lifestyle factors such as diet and physical activity may have changed over the follow-up period, and data on some variables were missing. These issues may introduce unmeasured confounding and bias the results. Moreover, the study covered a long time span. The participants had at least 3 years of occupational noise exposure at baseline, were followed for up to 9 years, and were required to have lipid data at both the 2016 and 2025 examinations. The reasons why some workers left their jobs were not investigated. Although the survivor effect was considered, the influence of other unmeasured factors cannot be excluded, and the overall health status of the sample may be subject to selection bias. A sensitivity analysis was performed to partially address this issue. Even in this relatively tolerant population, BHFTA was still associated with lipid deterioration, suggesting potential adverse effects of noise on lipid metabolism. Finally, the interpretation of BHFTA requires caution. BHFTA reflects accumulated auditory impairment arising from all noise sources, including non-occupational exposure, and is further influenced by age, ototoxic factors, and preexisting ear disease. It should therefore be regarded as an integrated marker of biologically effective noise dose and individual susceptibility rather than a direct measure of occupational noise exposure. Conversely, administratively recorded exposure duration is subject to measurement error because it does not capture actual noise levels or hearing protection use. The discordant findings between the two indicators may reflect the imprecision of administrative records, the limited specificity of BHFTA, or both, and cannot be separated without individual dosimetry data.

## Conclusions

In summary, among male workers with long-term occupational noise exposure, baseline cumulative noise exposure duration was significantly and positively associated with TG levels; its interaction with follow-up time showed a borderline negative trend, possibly reflecting the survivor effect, suggesting that the rate of lipid increase may slow with longer exposure duration. Higher BHFTA levels were significantly associated with a faster rate of lipid deterioration over time. Elevated hearing thresholds (accumulated auditory impairment) are not an isolated otological condition but rather an early warning signal of systemic cardiometabolic dysfunction. Future studies should integrate individual noise dose monitoring with multi-omics approaches to elucidate the molecular mechanisms underlying the interaction between auditory impairment and lipid metabolism.

## Data Availability

The raw data supporting the conclusions of this article will be made available by the authors, without undue reservation.
